# Histologic metabolic dysfunction–associated steatotic liver disease severity associated with a graded increase in peripheral artery disease risk

**DOI:** 10.1111/joim.70141

**Published:** 2026-07-26

**Authors:** Shuai Yuan, Fahim Ebrahimi, Jiangwei Sun, Hannes Hagström, Carole A. Marxer, Michael G. Clark, Marijana Vujkovic, Scott M. Damrauer, Jonas F. Ludvigsson

**Affiliations:** ^1^ Department of Surgery Perelman School of Medicine at the University of Pennsylvania Philadelphia Pennsylvania USA; ^2^ Department of Medical Epidemiology and Biostatistics Karolinska Institutet Stockholm Sweden; ^3^ Unit of Cardiovascular and Nutritional Epidemiology Institute of Environmental Medicine Karolinska Institutet Stockholm Sweden; ^4^ Corporal Michael J. Crescenz VA Medical Center Philadelphia Pennsylvania USA; ^5^ Department of Gastroenterology and Hepatology University Digestive Health Care Center Basel – Clarunis Basel Switzerland; ^6^ Division of Hepatology Department of Upper GI Diseases Karolinska University Hospital Stockholm Sweden; ^7^ Department of Medicine, Huddinge Karolinska Institutet Stockholm Sweden; ^8^ Department of Genetics University of Pennsylvania Perelman School of Medicine Philadelphia Pennsylvania USA; ^9^ Department of Pediatrics Örebro University Hospital Örebro Sweden; ^10^ Division of Digestive and Liver Disease Department of Medicine Columbia University Medical Center New York New York USA

**Keywords:** cohort, fatty, liver, peripheral artery diseases

Dear Editor,

Metabolic dysfunction–associated steatotic liver disease (MASLD, previously called NAFLD) affects approximately 30% of adults and is linked to cardiometabolic comorbidity [1]. MASLD has been associated with a higher risk of peripheral artery disease (PAD) [2]; however, few studies have evaluated risk across histologic severity stages [3]. Using a nationwide cohort of patients with biopsy‐verified MASLD, we quantified the risk of incident PAD overall and by MASLD severity.

The current study was based on the ESPRESSO study, a nationwide population‐based cohort containing liver histopathology from 28 Swedish pathology departments [4]. For each liver biopsy report, laboratories provided the personal identity number, biopsy date, liver topography (T56), and SNOMED morphology codes, and, when available, free text. ESPRESSO was linked to nationwide registers capturing demographics, level of education, comorbidities, prescribed medications, inpatient and hospital‐based outpatient care, and mortality. The study was approved by the Stockholm Regional Ethics Authority; informed consent was waived due to the register‐based nature of the study.

Individuals with MASLD were identified using SNOMED codes, international classification of disease (ICD) codes, and biopsy‐report text, restricting inclusion to adults (≥18 years) with a liver biopsy. Follow‐up began at biopsy or at the first relevant ICD code (index date). We excluded individuals with competing liver diseases, alcohol misuse, prior liver transplantation, or emigration before index to decrease misclassification. MASLD was classified as simple steatosis, MASH without fibrosis, non‐cirrhotic fibrosis, or cirrhosis. Each MASLD case was matched to up to five general‐population references (sex, age, county, and calendar year) and additionally compared with full siblings without the diagnosis of MASLD to account for intrafamilial confounding.

PAD was defined according to ICD‐codes or relevant procedure codes [5]. Metabolic conditions (obesity, diabetes, hypertension, and dyslipidemia) and chronic obstructive pulmonary disease (proxy for severe smoking) were identified from the National Patient Register using ICD codes, supplemented by Prescribed Drug Register data. Healthcare visits 6–24 months prior to index date captured healthcare utilization.

We examined incident PAD by MASLD presence and histologic severity, comparing MASLD patients with matched population comparators. Follow‐up ran from MASLD index biopsy to first PAD, death, emigration, or December 31, 2023. We computed incidence rate differences per person‐years and used Cox models conditioned on matching factors, with adjustment for education, metabolic conditions, and healthcare use. Sensitivity analyses included sibling comparisons and Fine‐Gray competing‐risk models. Proportionality assumption of Cox regression was tested by Schoenfeld residuals and found to be satisfied (*p* > 0.05). All analyses were performed using R version 4.4.1.

The analysis included 14,801 patients with MASLD matched to 68,945 general‐population comparators and 6865 patients with MASLD compared with 13,273 full siblings. During a median follow‐up of 16.5 years, we identified 376 incident PAD events among patients with MASLD (1.8 per 1000 person‐years) and 1124 events among comparators (0.9 per 1000 person‐years). The absolute rate difference was 0.8 per 1000 person‐years, corresponding to one additional PAD event per 168 patients with MASLD over 10 years. After multivariable adjustment, MASLD was associated with a 54% higher risk of incident PAD versus general population comparators (adjusted hazard ratio [aHR] 1.54; 95% CI, 1.33–1.79). Risk generally increased with histologic severity: aHR 1.47 (95% CI, 1.21–1.78) for simple steatosis, 1.59 (95% CI, 0.97–2.59) for MASH without fibrosis, and 2.17 (95% CI, 1.47–3.19) for non‐cirrhotic fibrosis, but slightly attenuated for individuals with cirrhosis (aHR 1.40; 95% CI, 0.81–2.43) (Fig. 1). Findings were consistent in full‐sibling comparisons (Fig. 1) and in competing‐risk analyses accounting for death.

**Fig. 1:**
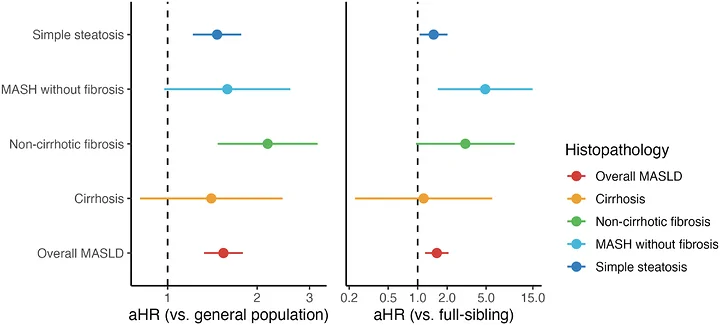
Adjusted hazard ratio (HR) of peripheral artery disease among individuals with metabolic dysfunction–associated steatotic liver disease (MASLD). The associations were conditioned on matching factors and additionally adjusted for education, country of birth, obesity, Type 2 diabetes, dyslipidemia, hypertension, chronic obstructive pulmonary disease after 40 years of age, and healthcare visits 6–24 months pre‐index.

Our findings are consistent with prior studies reporting higher PAD risk among individuals with MASLD [3] and extend the evidence by using biopsy verification and histologic staging. The monotonic increase from simple steatosis to non‐cirrhotic fibrosis supports a dose–response relation and argues against major residual confounding, further reinforced by concordant sibling comparisons. Biologically, MASLD—particularly fibrotic disease—may promote PAD through systemic insulin resistance, atherogenic dyslipidemia, chronic low‐grade inflammation, endothelial dysfunction, and prothrombotic pathways [3].

Limitations include selection bias from the biopsy‐based population, lack of longitudinal assessment of MASLD severity or noninvasive fibrosis measures such as FIB‐4 or elastography, differential comorbidity ascertainment from unequal healthcare contact, potential exposure misclassification due to unrecognized alcohol‐related liver disease, and limited precision for cirrhosis‐specific estimates, where competing mortality and liver‐related events may attenuate observed PAD incidence.

In conclusion, MASLD was associated with a higher risk of incident PAD that gradually increased with histologic severity, although the absolute excess risk was modest.

## Author contributions

**Hannes Hagström**: Methodology; validation; investigation; writing—review and editing; supervision; conceptualization. **Marijana Vujkovic**: Methodology; writing—review and editing; validation; investigation. **Jiangwei Sun**: Methodology; validation; investigation; writing—review and editing; conceptualization. **Shuai Yuan**: Conceptualization; methodology; software; data curation; investigation; formal analysis; project administration; visualization; writing—original draft; writing—review and editing. **Fahim Ebrahimi**: Methodology; investigation; validation; writing—review and editing; conceptualization. **Michael G. Clark**: Methodology; validation; writing—review and editing; investigation. **Carole A. Marxer**: Methodology; validation; writing—review and editing; investigation. **Scott M. Damrauer**: Methodology; validation; writing—review and editing; investigation; conceptualization; supervision. **Jonas F. Ludvigsson**: Conceptualization; methodology; data curation; supervision; resources; project administration; validation; writing—review and editing; funding acquisition; writing—original draft; investigation.

## Conflict of interest statement

S.M.D. receives personal consulting fees from Tourmaline Bio, research support from RenalytixAI, and in‐kind research support from Novo Nordisk and Amgen, all outside the scope of the current project. J.F.L. has received financial support from Merck/MSD for a study on inflammatory bowel disease and fibrosis and for developing a paper reviewing national healthcare registers in China. J.F.L. also has an ongoing research collaboration on celiac disease and chronic liver disease with Takeda. Earlier support includes a grant from Janssen for an unrelated study on behalf of the Swedish IBD quality register (SWIBREG). H.H.’s institutions have received research funding from Astra Zeneca, EchoSens, Gilead, Intercept, MSD, Novo Nordisk, Takeda, and Pfizer. He has served as consultant, speaker, or on advisory boards for Astra Zeneca, Boehringer Ingelheim, Bristol Myers‐Squibb, GSK, Echosens, Ipsen, MSD, and Novo Nordisk and has been part of hepatic events adjudication committees for Arrowhead, Boehringer Ingelheim, KOWA, and GW Pharma.

## References

[1] Younossi ZM , Golabi P , Paik JM , Henry A , Van Dongen C , Henry L . The global epidemiology of nonalcoholic fatty liver disease (NAFLD) and nonalcoholic steatohepatitis (NASH): a systematic review. Hepatology. 2023;77:1335–1547. 10.1097/HEP.0000000000000004. 36626630 PMC10026948

[2] Liu Y , Wang J , Jin R , Xu Z , Zhao X , Li Y , et al. Associations of metabolic dysfunction‐associated fatty liver disease with peripheral artery disease: prospective analysis in the UK Biobank and ARIC study. J Am Heart Assoc. 2024;13:e035265. 10.1161/JAHA.124.035265. 39547959 PMC11681420

[3] Yuan S , Damrauer SM , Larsson SC . Alcohol, liver disease, and peripheral arterial disease: epidemiology, mechanisms, and clinical implications. Arterioscler Thromb Vasc Biol. 2025;45:1493–1504. 10.1161/ATVBAHA.125.322136. 40501384 PMC12239223

[4] Ludvigsson JF , Lashkariani M . Cohort update: ESPRESSO (Epidemiology Strengthened by Histopathology Reports in Sweden). Clin Epidemiol. 2025;17:193–196. 10.2147/CLEP.S499859. 40027403 PMC11871922

[5] Yuan S , Damrauer SM , Håkansson N , Åkesson A , Larsson SC . A prospective evaluation of modifiable lifestyle factors in relation to peripheral artery disease risk. Eur J Vasc Endovasc Surg. 2022;64:83–91. 10.1016/j.ejvs.2022.04.004. 35472447

